# Ultrasound-Guided Peristyloid Hydrodissection for Eagle Syndrome With 5% Dextrose: A Technical Note Integrating Computed Tomography-Defined Anatomy, Doppler Vascular Mapping, and Orthogonal Sonographic Guidance

**DOI:** 10.7759/cureus.116155

**Published:** 2026-09-12

**Authors:** Yonghyun Yoon, Dongyeun Sung, Jonghyeok Lee, Seungbeom Kim, Youngmo Kim, Teinny Suryadi, Anwar Suhaimi, King Hei Stanley Lam

**Affiliations:** 1 Orthopedics, International Academy of Musculoskeletal Medicine, Hong Kong, HKG; 2 Orthopedics, International Academy of Regenerative Medicine, Incheon, KOR; 3 Orthopedic Surgery, Hallym University Kangnam Sacred Heart Hospital, Seoul, KOR; 4 Orthopedic Surgery, Incheon Terminal Orthopedic Clinic, Incheon, KOR; 5 Neurosurgery, Himchannamu Neurosurgery Clinic, Daegu, KOR; 6 Neurosurgery, Bareun Neurosurgery Clinic, Cheongju, KOR; 7 Pain Medicine, Miso Pain Clinic, Suwon, KOR; 8 Orthopedics, Incheon Terminal Orthopedic Clinic, Incheon, KOR; 9 Physical Medicine and Rehabilitation, Medistra Hospital, South Jakarta, IDN; 10 Physical Medicine and Rehabilitation, Synergy Clinic, Jakarta, IDN; 11 Physical Medicine and Rehabilitation, Hermina Hospital Podomoro, Jakarta, IDN; 12 Rehabilitation Medicine, University Malaya Medical Centre, Lembah Pentai, MYS; 13 Clinical Research, International Academy of Regenerative Medicine, Incheon, KOR; 14 Medicine, The Chinese University of Hong Kong, New Territories, HKG; 15 Medicine, The University of Hong Kong, Pok Fu Lam, HKG

**Keywords:** 5% dextrose, 5% dextrose water, dysphagia, eagle syndrome, hydrodissection, peristyloid dissection, styloid process, ultrasound-guided injection

## Abstract

Eagle syndrome is a symptomatic disorder associated with elongation or abnormal orientation of the styloid process and/or ossification of the stylohyoid complex. Clinical manifestations depend on the spatial relationship between the styloid complex and adjacent neural, muscular, fascial, and vascular structures. We describe an ultrasound-guided peristyloid hydrodissection technique using 5% dextrose in water (D5W) that integrates preprocedural computed tomography (CT), sequential landmark-based sonographic localization, live-dual imaging, Doppler vascular mapping, and orthogonal short- and long-axis confirmation. A 24-year-old man presented with more than six months of predominantly right-sided pharyngeal foreign-body sensation and dysphagia, accompanied by cervical pain, headache, and intermittent dizziness. The right styloid process measured 37 mm on CT. D5W hydrodissection was performed once weekly for six weeks. In this index case, 10 mL was delivered to the superior peristyloid tissue plane and 10 mL to the inferior plane at each session, with visible sonographic tissue-plane separation as the procedural endpoint. This 20 mL volume was specific to the index case and should not be extrapolated to future patients. The patient reported gradual improvement in pharyngeal symptoms during the treatment course; pain decreased from 7/10 to 3/10 on the numeric rating scale after six sessions, with maintained subjective benefit at three-month follow-up and no adverse events. This report presents a reproducible image-guided procedural framework rather than evidence of treatment efficacy. Cadaveric validation and prospective studies are required to define injectate distribution, optimal dosing, safety, and clinical effectiveness.

## Introduction

Eagle syndrome is characterized by symptoms attributable to an elongated styloid process or ossified stylohyoid complex. Typical manifestations include pharyngeal foreign-body sensation, dysphagia or odynophagia, cervicofacial pain, and otalgia; headache and less common neurovascular symptoms may also occur. Styloid elongation alone does not establish symptomatic Eagle syndrome because clinical expression depends on the length, angulation, and relationship of the styloid complex to adjacent soft-tissue and neurovascular structures [1,2].

Three-dimensional CT is useful for defining styloid morphology and orientation, particularly when skull-base or vascular relationships are clinically relevant [1,2]. Ultrasound provides complementary real-time information: the styloid process can be dynamically traced, regional vessels can be mapped with Doppler, and needle position and injectate spread can be observed during intervention. Ultrasound-guided peristyloid steroid injection has been reported for Eagle syndrome, but a standardized hydrodissection workflow integrating multiaxial sonoanatomy and continuous vascular mapping has not been described in detail [3].

The purpose of this technical report is to describe a structured ultrasound-guided peristyloid hydrodissection technique using 5% dextrose in water (D5W) and to illustrate its application in a young adult with imaging-confirmed styloid elongation and persistent pharyngeal symptoms. A second objective is to emphasize the layered anatomy of the styloid region. Simplified schematics may imply that multiple cranial nerves and vessels occupy a single peristyloid compartment, whereas procedural planning requires recognition of distinct extracranial courses and patient-specific vascular relationships. Our previous cadaveric validation study of ultrasound-guided access to the facial nerve trunk similarly demonstrated the value of combining musculoskeletal and vascular landmarks rather than relying on a single osseous landmark [4].

## Technical report

Case presentation and preprocedural assessment

A 24-year-old man presented with predominantly right-sided symptoms persisting for more than six months. His principal complaints were a persistent pharyngeal foreign-body sensation and dysphagia, accompanied by cervical pain and headache. Intermittent dizziness was a secondary complaint and worsened with neck rotation. Baseline pain intensity was 7/10 on a numeric rating scale (NRS). He had no history of tonsillectomy or relevant cervical trauma. The right styloid process was palpable through the tonsillar fossa, and palpation reproduced the characteristic foreign-body sensation. Cranial nerve examination was intact. Gaze-evoked horizontal nystagmus was noted without focal cranial nerve deficits. Previous treatment with analgesic and anti-inflammatory medication and physical therapy at another institution had not produced meaningful improvement. A prior magnetic resonance imaging study performed elsewhere was unrevealing. Plain radiography and CT were reviewed during the present evaluation, and styloid elongation was assessed on both modalities. The right styloid process measured 37 mm on CT. Three-dimensional CT reconstruction was used to evaluate its orientation relative to the mandible, mastoid region, and upper cervical osseous landmarks (Figure 1).

**Figure 1:**
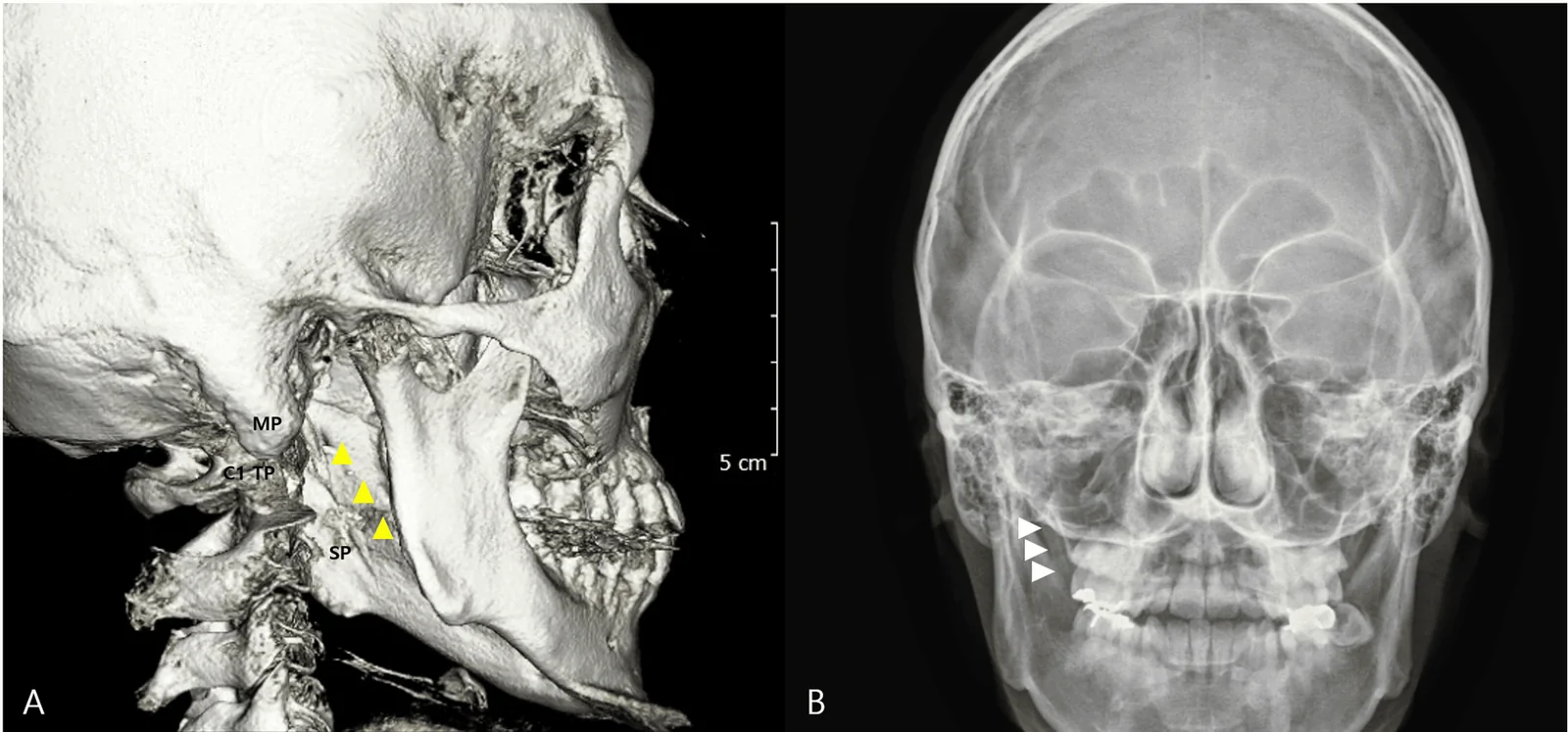
Radiographic and computed tomography findings of the elongated right styloid process. (A) Three-dimensional computed tomography reconstruction demonstrating the elongated right styloid process (yellow arrowheads) and its anatomical relationship with the mastoid process and the C1 transverse process; (B) Frontal radiograph demonstrating the elongated right styloid process (white arrowheads). MP, mastoid process; C1 TP, C1 transverse process; SP, styloid process.

Angiographic or dynamic vascular imaging was not performed; therefore, a concomitant vascular Eagle phenotype could not be formally excluded. The indication for the present intervention was based primarily on the dominant classic pharyngeal symptom complex, particularly persistent foreign-body sensation and dysphagia, rather than on the intermittent dizziness.

Sonoanatomic localization and vascular mapping

The patient was placed in the lateral decubitus position with the symptomatic side facing upward and the head supported in a neutral position. A high-frequency linear transducer was used. Examination began by identifying the mastoid region and the C1 transverse process as reproducible osseous landmarks for orientation within the deep lateral neck.

The transducer positions and scanning workflow are illustrated schematically on a three-dimensional CT reconstruction (Figure 2).

**Figure 2:**
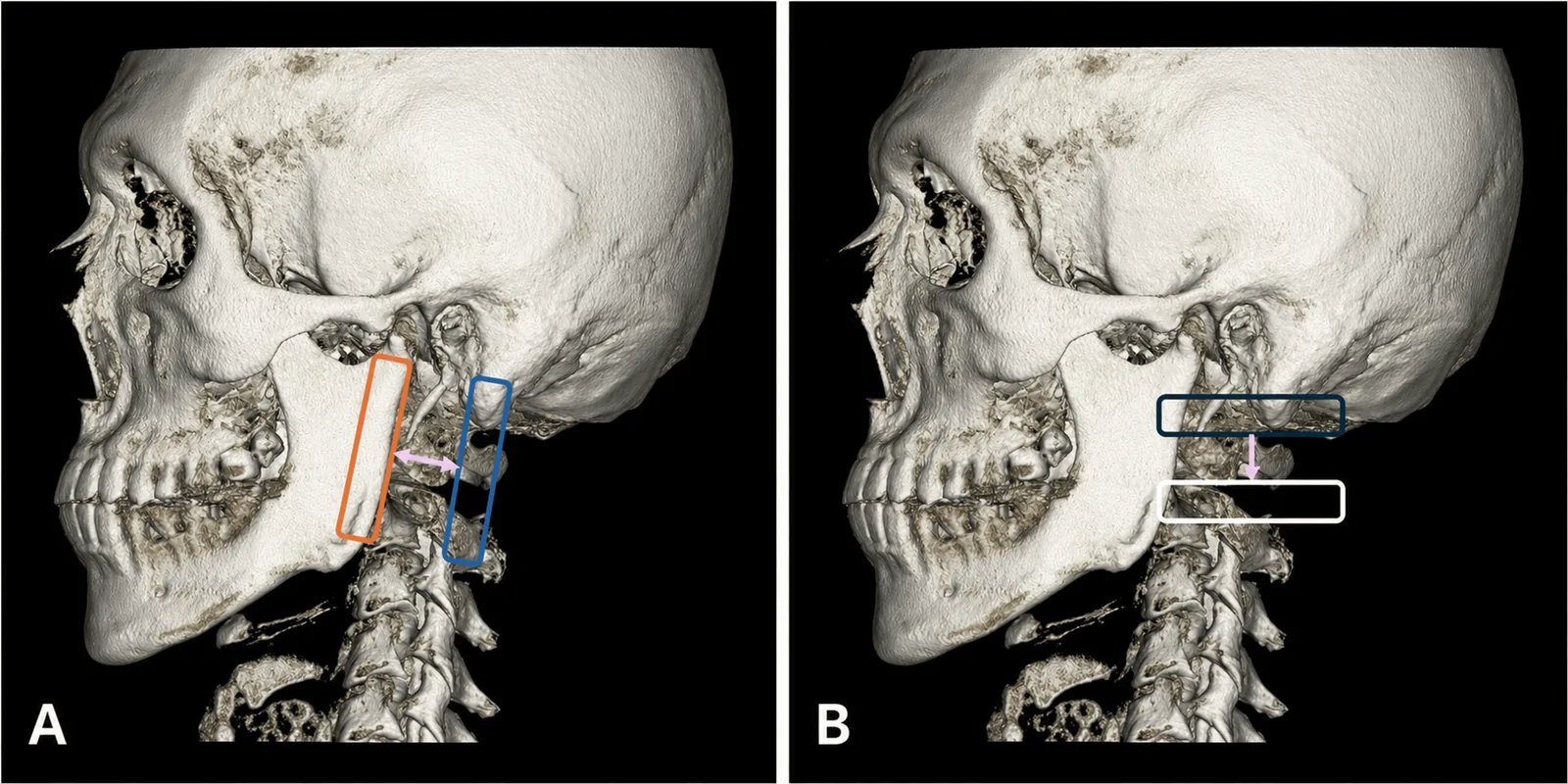
Schematic transducer positioning for ultrasound-guided localization of the styloid region (A) Three-dimensional CT reconstruction with schematic transducer overlays demonstrating posterior-to-anterior probe translation. The orange and blue rectangles indicate sequential transducer positions, and the arrow indicates the direction of probe movement. (B) Three-dimensional CT reconstruction demonstrating cranial-to-caudal probe translation. The dark blue and white rectangles indicate sequential transducer positions, and the arrow indicates the direction of probe movement. The transducer overlays are schematic and indicate approximate probe positions and scanning directions.

Panel A demonstrates the posterior-to-anterior transducer translation used to approach and dynamically trace the styloid region, whereas panel B demonstrates the cranial-to-caudal transducer translation. The colored rectangles indicate approximate transducer positions, and the arrows indicate the direction of probe movement. These overlays are schematic and are intended to illustrate transducer positioning rather than soft-tissue anatomy.

Dynamic sonographic localization was then performed using the transducer movements illustrated schematically in Figure 2. Cranial-to-caudal scanning (Figure 2B; Video 1) from the mastoid and upper cervical reference region allowed the osseous contour of the styloid process to be followed across consecutive views.

**Video 1:** Cranial-to-caudal localization of the styloid process Dynamic ultrasound demonstrates sequential cranial-to-caudal probe translation from the mastoid and upper cervical reference region toward the styloid process, allowing the osseous contour to be followed across consecutive views. SCM, sternocleidomastoid muscle; PBDM, posterior belly of the digastric muscle; LC, longissimus capitis; LS, levator scapulae; OA, occipital artery; ICA, internal carotid artery; ECA, external carotid artery; C1 TP, C1 transverse process; C2 TP, C2 transverse process; VA, vertebral artery; SP, styloid process; IOC, inferior oblique capitis muscle.

Posterior-to-anterior scanning (Figure 2A; Video 2) was subsequently performed to reassess the changing relationship of the styloid process to adjacent muscular and vascular structures.

**Video 2:** Posterior-to-anterior scanning of the styloid region Posterior-to-anterior probe translation demonstrates the changing relationship of the styloid process to adjacent muscular and vascular structures. MP, mastoid process; PBDM, posterior belly of the digastric muscle; C1 TP, C1 transverse process; SP, styloid process.

Posterior-to-anterior scanning was subsequently performed to demonstrate the changing relationship of the styloid process to adjacent muscular and vascular structures (Video 2). Across both scanning sequences, the styloid process was identified as a hyperechoic cortical interface with posterior acoustic shadowing, and its relationship to the C1 transverse process, posterior belly of the digastric muscle, styloid-associated musculature, parotid gland, and surrounding vascular structures was reassessed dynamically.

Before needle advancement, color Doppler was activated and maintained during live-dual imaging. The posterior auricular and occipital arteries were identified in the posterolateral scanning field, and deeper vascular anatomy was reassessed as the transducer position changed. A needle trajectory was selected only when a vessel-free path could be maintained under direct visualization (Figure 3).

**Figure 3:**
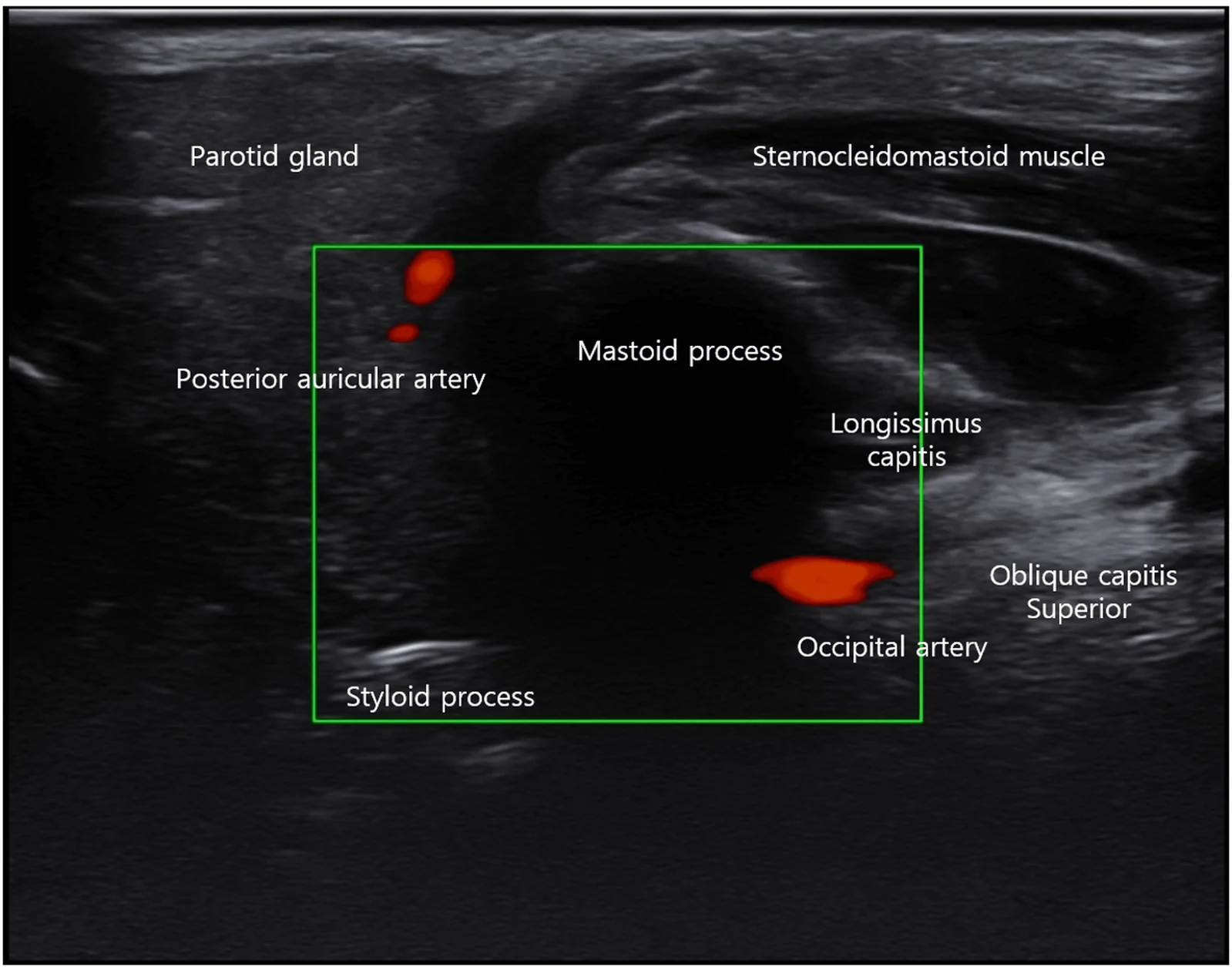
Doppler vascular mapping of the posterolateral styloid region Representative color Doppler imaging demonstrates the styloid and mastoid processes with the adjacent posterior auricular and occipital arteries. The parotid gland, sternocleidomastoid muscle, longissimus capitis muscle, and obliquus capitis superior muscle are shown as additional sonographic landmarks. Doppler assessment was performed before and during needle advancement to define an individualized vessel-free working corridor.

Because vascular relationships in the upper cervical region vary among individuals, Doppler mapping was incorporated into procedural planning rather than relying on presumed textbook anatomy.

Ultrasound-guided peristyloid hydrodissection

After sterile preparation, a 23 gauge, 6 cm needle was introduced in-plane from posterior to anterior under continuous ultrasound visualization. During the initial phase, live-dual imaging was used to maintain simultaneous anatomical orientation. Needle advancement was hydrodissection-assisted rather than performed as a dry-needle approach. Small incremental aliquots of D5W were injected ahead of the needle tip before the final peristyloid target plane was reached, creating a fluid interface and progressively separating adjacent soft-tissue and vascular structures from the intended trajectory. Further advancement was undertaken only after extravascular injectate spread and opening of the tissue plane had been directly confirmed. Posterior-to-anterior in-plane hydrodissection under live-dual guidance is demonstrated in Video 3.

**Video 3:** Posterior-to-anterior in-plane peristyloid hydrodissection using live-dual imaging The video demonstrates posterior-to-anterior in-plane advancement of the needle toward the peristyloid tissue plane while live-dual imaging is maintained for simultaneous anatomical orientation. Incremental 5% dextrose in water injection is performed ahead of the advancing needle tip under continuous visualization to open the tissue plane and confirm extravascular tissue-plane separation before further needle advancement. HD, hydrodissection; SCM, sternocleidomastoid muscle; PBDM, posterior belly of the digastric muscle; LC, longissimus capitis; C1 TP, C1 transverse process; IOC, inferior oblique capitis muscle; SP, styloid process.

Color Doppler was used repeatedly to reassess vascular structures adjacent to the needle trajectory and target region.

No aspiration maneuver was incorporated into the index procedure. Instead, needle advancement was hydrodissection-assisted from an early stage, with small aliquots of D5W injected ahead of the needle tip to establish an extravascular fluid plane before further advancement. Needle position and procedural safety were assessed by continuous visualization of the needle tip, repeated Doppler mapping of adjacent vascular structures, incremental low-pressure injection, and direct visualization of extravascular injectate spread. Further advancement was performed only after an appropriate tissue plane had been opened and confirmed sonographically. If the needle tip became indistinct, vascular flow entered the intended trajectory, unexpected resistance was encountered, or extravascular spread could not be clearly confirmed, advancement or injection was stopped and the pathway was reassessed. Nevertheless, given the vascular density of the upper cervical region, we recommend aspiration before injection and after significant needle repositioning as an additional safety check in future applications of this technique.

D5W was used without local anesthetic or corticosteroid. The procedural endpoint was sonographic rather than tactile: successful hydrodissection was defined by visible expansion of the targeted soft-tissue interface with progressive extravascular spread of injectate.

After the initial posterior-to-anterior in-plane hydrodissection, live-dual imaging was discontinued and the transducer was rotated to align with the long axis of the styloid process. Using this longitudinal view, the needle was then positioned sequentially above and below the styloid process, and D5W was administered to the superior and inferior peristyloid tissue planes (Video 4). 

**Video 4:** Ultrasound-guided hydrodissection along the superior aspect of the styloid process After completion of the posterior-to-anterior hydrodissection, Live dual imaging was discontinued and the transducer was rotated to align with the long axis of the styloid process. Under real-time ultrasound guidance, 5% dextrose in water was injected along both the superior and inferior aspects of the styloid process to achieve hydrodissection around the target region. The white arrowhead indicates the needle. HD, hydrodissection; SP, styloid process.

At each session, 10 mL was delivered to the superior plane and 10 mL to the inferior plane, for a total volume of 20 mL. Injection into each plane was performed incrementally under real-time ultrasound guidance, with visible tissue-plane separation used as the procedural endpoint. This volume reflects the protocol actually used in the index case (volume-related safety considerations are presented in the discussion).

The patient underwent one session per week for six consecutive weeks. This once-weekly interval and six-session course were selected empirically, based on the treating physicians' clinical experience with serial hydrodissection protocols and on the patient's incremental symptomatic response after each session, rather than on a validated Eagle syndrome-specific treatment-frequency protocol. The six-session schedule, like the injectate volume discussed above, was a case-specific procedural parameter rather than an established treatment-frequency regimen for Eagle syndrome.

Clinical course

Across six once-weekly treatments to the right peristyloid region, the patient reported gradual improvement in the foreign-body sensation, dysphagia, cervical pain, and headache during the treatment course. Dizziness was not used as a primary treatment-response endpoint. After the sixth session, pain intensity decreased from 7/10 to 3/10 on the NRS, and the patient's subjective global improvement was maintained at three-month follow-up. No immediate or delayed adverse events were reported. After each procedure, the patient was observed for 30 minutes with assessment of vital signs and monitoring for respiratory symptoms, dizziness, and new neurologic or systemic symptoms. Written informed consent was obtained for the procedure and for publication of clinical information, images, and videos. The single-patient technical report was considered exempt from formal institutional review board review under the applicable institutional policy.

## Discussion

The principal technical concept of this report is that the styloid process should be approached as part of a three-dimensional, multilayered region rather than as an isolated bony target. The classic Eagle syndrome phenotype is dominated by pharyngeal and cervicofacial symptoms, including foreign-body sensation, dysphagia, and pain, which may reflect irritation of neural, muscular, or fascial structures surrounding the styloid complex [1,2]. Vascular relationships remain important for procedural safety and differential diagnosis but were not the primary clinical focus in this case.

Anatomical oversimplification becomes clinically relevant when multiple cranial nerves are depicted as if they traverse a single uniform peristyloid compartment. The facial nerve illustrates this limitation: after exiting the stylomastoid foramen, the extracranial trunk courses within a region defined by the mastoid, posterior belly of the digastric muscle, parotid tissue, and adjacent vessels. In our prior cadaveric validation study, ultrasound-guided access to the facial nerve trunk was developed using integrated musculoskeletal and vascular landmarks and confirmed by post-injection anatomical dissection [4]. That study supports the broader procedural principle applied here: deep craniofacial interventions should be planned using layered, patient-specific sonoanatomy rather than a simplified relationship to a single osseous landmark.

The present technique also differs from previously described styloid-region interventions. Maher and Shankar reported ultrasound-guided peristyloid steroid injection for Eagle syndrome [3], whereas styloid-based ultrasound approaches have also been used to guide glossopharyngeal nerve blockade for glossopharyngeal neuralgia [5]. More recently, a 2026 Cureus case report described repeated office-based ultrasound-guided glossopharyngeal nerve blocks for Eagle syndrome, with symptomatic benefit lasting approximately three months [6]. In contrast, the current technique uses D5W to create visible separation of superior and inferior peristyloid soft-tissue interfaces. The intended target is the tissue plane adjacent to the styloid process rather than focal deposition around a named cranial nerve. This should not be interpreted as osseous decompression, shortening of the styloid process, or permanent enlargement of the styloid-C1 interval.

D5W is established as an injectate for ultrasound-guided nerve hydrodissection. A mechanistic and clinical review summarized its use for separating nerves from surrounding fascia or adjacent structures [7], and randomized evidence in carpal tunnel syndrome demonstrated clinical benefit from ultrasound-guided perineural D5W compared with corticosteroid injection [8]. These data support the general use of D5W as a hydrodissection injectate but do not establish efficacy, dose, or treatment frequency for Eagle syndrome.

Cervical applications of D5W hydrodissection have also been reported. Lam and colleagues described bilateral vagus nerve hydrodissection with D5W without local anesthetic for chronic multisite pain and autonomic dysfunction [9]. A subsequent Cureus technical report detailed simultaneous hydrodissection of the cervical sympathetic chain and vagus nerves, emphasizing anatomical correlation, real-time ultrasound visualization, and slow injection [10]. These reports demonstrate technical feasibility of D5W hydrodissection in the cervical region; they do not imply that vagal or sympathetic-chain interventions are equivalent to a peristyloid procedure or that they address the same anatomical pain generator.

The 37 mm right styloid process was elongated by commonly used imaging thresholds, but the diagnostic rationale rested on concordance between the osseous abnormality, dominant pharyngeal symptoms, and reproduction of the foreign-body sensation by tonsillar fossa palpation rather than length alone [1,2]. Three-dimensional CT defined osseous morphology, while ultrasound provided dynamic information on soft tissues, vessels, needle position, and injectate spread. The prior outside-hospital magnetic resonance imaging study was unrevealing. The patient's intermittent, rotation-provoked dizziness raises a genuine diagnostic dilemma. Positional dizziness in the setting of an elongated styloid process can reflect dynamic compression of the internal carotid artery, internal jugular vein, or vertebral artery during head rotation, in addition to more common peripheral vestibular causes; the two are not mutually exclusive, and a coexisting vascular Eagle phenotype can occur alongside the classic pharyngeal presentation that was our primary treatment target. Because dedicated angiographic or dynamic vascular imaging was not performed, a vascular contribution cannot be formally excluded in this case, and this is an important diagnostic limitation. The present intervention was directed primarily at the dominant classic pharyngeal symptom complex, and dizziness was not used as a treatment-response endpoint. We now specify more concretely when vascular imaging should be pursued before serial peristyloid intervention: any patient with rotation-triggered or positional dizziness, presyncope, syncope, pulsatile tinnitus, transient visual disturbance, or focal neurologic symptoms should undergo computed tomography angiography or venography (CTA/CTV; guided by suspected arterial versus venous involvement) with dynamic head-rotation imaging or Doppler assessment prior to treatment, so that a dynamic neurovascular conflict is identified and, if present, managed accordingly before-or instead of-peristyloid hydrodissection.

The relationship between the styloid process and the C1 transverse process remains relevant to the differential diagnosis. Compression of the internal jugular vein between these structures has been described in styloidogenic jugular venous compression syndromes [11,12], and a recent review has further emphasized the broader vascular spectrum of Eagle syndrome [13]. The present hydrodissection technique does not alter fixed osseous dimensions. Any biological or mechanical effect should therefore be framed as soft-tissue interface separation rather than structural decompression of a bony corridor. Accordingly, peristyloid hydrodissection should not be considered a treatment for fixed carotid or jugular compression caused by an osseous styloid-C1 relationship.

The 20 mL total volume used at each session represents the case-specific protocol applied in this patient; clinicians should not extrapolate this volume to future cases in the absence of validated dosing data. There is currently no validated Eagle syndrome-specific D5W hydrodissection volume or treatment frequency. Because the peristyloid region contains closely adjacent neurovascular structures and its tolerance to injectate volume has not been established, future procedures should favor the minimum volume necessary to achieve clearly visible sonographic tissue-plane separation, delivered incrementally, slowly, and at low pressure. The absence of adverse events in this single case does not establish the safety of the 20 mL volume. Prospective dose-ranging and anatomical validation studies are needed to determine whether lower volumes and fewer or symptom-guided sessions can achieve comparable procedural and clinical outcomes.

Several limitations require emphasis. First, this single-patient technical report cannot establish treatment efficacy, and the principal outcome was patient-reported global improvement rather than a prospectively defined swallowing- or globus-specific instrument. Second, the exact distribution of D5W cannot be established by ultrasound alone; cadaveric dye validation would be valuable for defining the structures reached by superior and inferior peristyloid injection. Third, dedicated angiographic or dynamic vascular imaging was not performed despite rotation-related dizziness; therefore, a concomitant vascular Eagle phenotype could not be excluded. Future patients with positional neurovascular symptoms should undergo appropriate vascular assessment before serial peristyloid intervention. Fourth, aspiration was not performed in the index procedure. Although the technique used hydrodissection-assisted needle advancement with continuous needle-tip visualization, Doppler vascular mapping, incremental low-pressure injection, and direct confirmation of extravascular injectate spread, we recommend negative aspiration before injection and after significant needle repositioning as an additional safety check in future applications. Fifth, the safety and optimal volume of peristyloid D5W injection remain undefined. Finally, surgical styloidectomy remains the definitive structural treatment for appropriately selected patients with refractory Eagle syndrome, and contemporary surgical literature continues to support styloidectomy as an effective option [14]. The technique described here should, therefore, be interpreted as a preliminary, minimally invasive procedural framework requiring further anatomical and clinical validation.

## Conclusions

This technical report describes a structured ultrasound-guided approach to D5W peristyloid hydrodissection in a patient with symptomatic right styloid elongation and dominant pharyngeal foreign-body sensation and dysphagia. The technique integrates preprocedural radiography and CT, dynamic styloid tracing, live-dual imaging, Doppler vascular mapping, continuous needle-tip visualization, orthogonal confirmation, and a sonographic tissue-separation endpoint. In this case, the dominant symptoms improved gradually during six weekly sessions and subjective global benefit was maintained at the three-month follow-up without reported adverse events. These observations do not establish efficacy. Cadaveric validation and prospective clinical studies are required to characterize injectate distribution, define dosing and treatment frequency, establish safety, and identify clinical phenotypes most likely to benefit.
